# In-Hospital Formula Feeding Hindered Exclusive Breastfeeding: Breastfeeding Self-Efficacy as a Mediating Factor

**DOI:** 10.3390/nu15245074

**Published:** 2023-12-12

**Authors:** Lu Liu, Yuju Wu, Xiannan Xian, Jieyuan Feng, Yuping Mao, Siva Balakrishnan, Ann M. Weber, Gary L. Darmstadt, Yunwei Chen, Sean Sylvia, Huan Zhou, Scott Rozelle

**Affiliations:** 1 Department of Health Behavior and Social Medicine, West China School of Public Health and West China Fourth Hospital, Sichuan University, No. 16 South Renmin Road 3 Section, Chengdu 610041, China; lu_liu@stu.scu.edu.cn (L.L.); yujuwu@scu.edu.cn (Y.W.); xianxiannan@stu.scu.edu.cn (X.X.); 2 Stanford Center on China’s Economy and Institutions, Freeman Spogli Institute for International Studies, Stanford University, Stanford, CA 94305, USA; cindyfn@stanford.edu (J.F.); rozelle@stanford.edu (S.R.); 3 Department of Communication Studies, College of Liberal Arts, California State University Long Beach, Long Beach, CA 90840, USA; yuping.mao@csulb.edu; 4 Department of Biostatistics, Epidemiology and Environmental Health, School of Public Health, University of Nevada, Reno, NV 89503, USA; sbalakrishnan@unr.edu (S.B.);; 5 Department Pediatrics, Stanford University School of Medicine, Stanford, CA 94305, USA; gdarmsta@stanford.edu; 6 Health Policy and Management, University of North Carolina at Chapel Hill, Chapel Hill, NC 27599, USA; ywchen@email.unc.edu (Y.C.); sysylvia@email.unc.edu (S.S.)

**Keywords:** breastfeeding, breastfeeding self-efficacy, in-hospital formula feeding, mediation

## Abstract

Breastfeeding self-efficacy (BSE), defined as a mother’s confidence in her ability to breastfeed, has been confirmed to predict the uptake of exclusive breastfeeding (EBF). Early experiences during the birth hospital stay, especially in-hospital formula feeding (IHFF), can impact both EBF and maternal breastfeeding confidence. Therefore, our objective was to examine the association between IHFF and EBF outcomes and investigate whether this association is influenced by BSE. The study included 778 infants from a larger cohort study conducted in 2021, with a one-year follow-up in rural areas of Sichuan Province, China. We used a causal mediation analysis to estimate the total effect (TE), natural direct (NDE), and nature indirect effects (NIE) using the paramed command in Stata. Causal mediation analyses revealed that IHFF was negatively associated with EBF (TE odds ratio = 0.47; 95% CI, 0.29 to 0.76); 28% of this association was mediated by BSE. In the subgroup analysis, there were no significant differences in the effects between parity subgroups, as well as between infant delivery subgroups. Our study found that IHFF hindered later EBF and that BSE mediated this association. Limiting the occurrence of in-hospital formula feeding or improving maternal breastfeeding self-efficacy is likely to improve exclusive breastfeeding outcomes.

## 1. Introduction

Exclusive breastfeeding (EBF), defined as providing only breast milk without any other food or liquids, benefits both infants aged 0–6 months and mothers [1]. Promoting greater uptake and duration of EBF has been associated with a lower risk of infant mortality [2], reduced overweight and obesity prevalence at 2 to 5 years old, lowered non-communicable disease risk [3], and improved cognition and educational outcomes [4,5]. EBF offers protective benefits for mothers as well, including a reduced breast and ovarian cancer risk and prolonging the period of lactational amenorrhea [6]. However, in 2022, global EBF rates were at only 48% and found to decline monthly for infants within the 0–6-month-old age group [7,8].

Early experiences during the birth hospital stay, such as formula feeding, can impact the likelihood of successful EBF later in life [9,10]. In-hospital formula feeding (IHFF) has been associated with shorter breastfeeding durations [11,12] and a 50% reduction in the likelihood of EBF [10,13]. Additionally, the introduction of formula milk in the neonatal period may disrupt maternal care patterns and milk production, impacting EBF success [10,14]. Formula-feeding newborns is a common practice in birth hospitals, with approximately one-quarter of U.S. hospitals providing formula supplementation to at least 50% of healthy newborns between 2009 and 2013 [15]. Studies conducted in Croatia [16] and Hong Kong [17] found that 62.8% and 82.5% of newborns received IHFF, respectively.

Identifying potential modifiable mediators between IHFF and EBF is crucial for designing effective breastfeeding interventions. Breastfeeding self-efficacy (BSE), defined as a mother’s confidence in her ability to breastfeed, has been confirmed by several studies to predict both the decision to breastfeed and the uptake of EBF [18,19]. These studies postulate that the strength of a mother’s BSE influences her breastfeeding behavior, such as the initiation and duration of breastfeeding [18]. Although the association between IHFF and BSE remains unclear, some research suggests that IHFF may reduce maternal breastfeeding confidence [12,13]. Therefore, in this study, we hypothesized that BSE mediates the association between IHFF and EBF outcomes.

Our study examined (1) whether IHFF is associated with both BSE and EBF outcomes; (2) whether BSE is associated with EBF outcomes; and (3) whether BSE mediates the associations between IHFF and EBF outcomes.

## 2. Materials and Methods

### 2.1. Study Design and Participants

This research is part of a larger cohort study conducted in the rural areas of Nanchong City in Sichuan Province. The research team followed a multistage cluster sampling protocol to select the study sample. The first step involved randomly selecting all four nationally designated poverty counties of Nanchong. Next, 20 townships were chosen randomly from each county, totaling 80 townships. We excluded townships a with population less than 10,000 and townships classified as non-farming townships. Finally, in each sampled township, all households with infants aged 0 to 5 months (or less than 183 days old) residing permanently within the township were included.

Baseline data were collected through face-to-face interviews conducted in July and August of 2021. The following groups were excluded from the sample: infants or young children with major diseases, mothers who suffered from a severe mental illness or intellectual disability, or participants with missing data variables. All remaining participants who gave their written informed consent prior to data collection were included in the study. Our final sample comprised 778 mother–child dyads. This study was approved by the Institutional Review Board at Sichuan University (protocol K2019046, approved on 15 July 2019). All study participants provided their informed consent prior to enrolment and understood that their participation was purely voluntary.

### 2.2. Measurements

#### 2.2.1. In-Hospital Formula Feeding (Exposure of Interest)

We defined IHFF as the practice of providing formula to newborns during their hospital stay after birth [12,13]. We asked the participating caregivers, “Was your baby given formula while in the hospital?” Responses indicating “yes” were coded as IHFF = 1, while responses indicating “no” were coded as IHFF = 0.

#### 2.2.2. Breastfeeding Self-Efficacy (Mediator of Interest)

We administered the Breastfeeding Self-Efficacy Scale-Short Form (BSES-SF) to assess the BSE of all participating caregivers [20]. BSES-SF is a widely used assessment tool that has undergone validation procedures to ensure its reliability and validity for measuring BSE [21]. The BSES-SF contains 14 items and uses a 5-point Likert scale to gauge a participant’s level of confidence (Table 1). The scale ranges from “1 = not confident at all” to “5 = always confident”, resulting in a total score range of 14 to 70. In our analysis, we used a dichotomous approach to categorize BSES-SF scores, utilizing the 50th percentile as the threshold (P50 = 48), which aligns closely to the threshold recommended by a study carried out in Japan [22].

#### 2.2.3. Exclusive Breastfeeding Outcomes

To determine if an infant was exclusively breastfed or not, we utilized a carefully designed questionnaire that collected data on infant and young child feeding activities over a 24 h period [1]. Participating caregivers were asked to recall every solid and liquid food item consumed by their infant in the 24 h prior to data collection. To prevent caregivers from forgetting, we designed our questionnaire according to WHO recommendations and asked for 24 h dietary recall of the infants’ consumption of breastmilk, breastmilk substitutes (e.g., infant formula or animal milk), plain water, non-milk liquids (e.g., juice or broth), and semi-solid or solid foods (e.g., porridge or rice). And we inserted the local names of these common foods into the recall. Infants falling within the age range of under 6 months, for whom the consumption of solely breast milk was reported during this timeframe, were categorized as being exclusively breastfed [1]. And the definition only considered the period after hospital discharge and did not consider in-hospital feeding [23].

#### 2.2.4. Covariates

**Figure 1:**
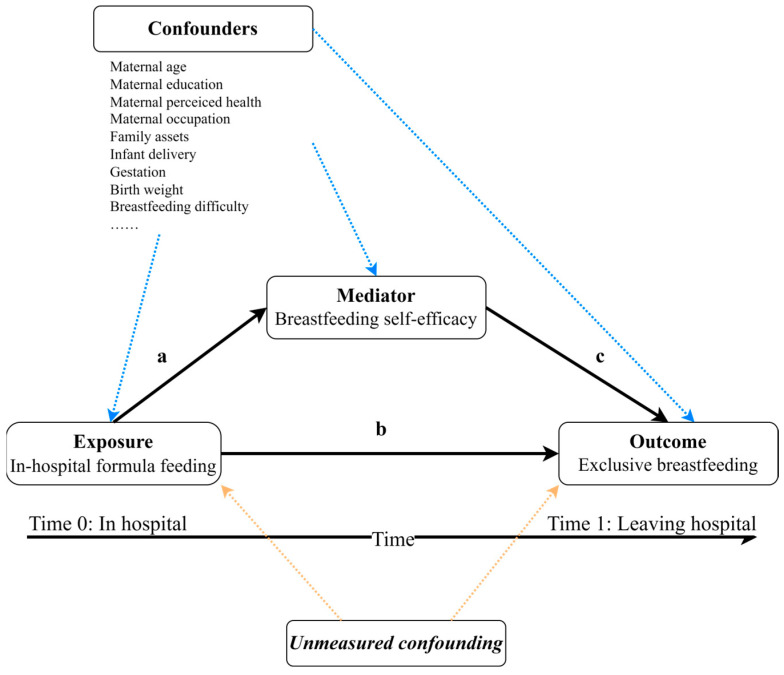
Model hypothesis of in-hospital formula feeding, exclusive breastfeeding, and breastfeeding self-efficacy with covariates. Indirect effects are generated through paths a + c, whereas direct effects are generated through path b.

To select appropriate covariates, we selected potential confounding variables from previous studies [11,12,13,23] and used the evidence synthesis for constructing directed acyclic graph (DAG) method to plot directed acyclic graphs using DAGitty v3.1 software. DAGs use the “backdoor criterion”, a mathematical ruleset, to determine which variables should be controlled for when estimating the effect of one on another [24]. In all adjusted model, we treated maternal age, maternal education, maternal perceived personal health, maternal occupation status, family assets, infant delivery, infant sex, gestation, birth weight, breastfeeding difficulties, pregnancy complications, and parity as covariates (Figure 1).

Family assets were assessed using a household asset index generated using a polychoric principal component analysis based on whether a household owned or had access to a water heater, washing machine, refrigerator, air conditioner, television, computer, motorcycle, and car or truck, which was then divided into two asset groups (low assets and high assets) [25]. Infant gestational week was determined from birth certificates. If an infant was born at less than 37 weeks gestation, they were classified as preterm; infants born at 37 weeks of gestation or more were classified as full term. Finally, infant birth weight was categorized as low birth weight (LBW) when an infant’s weight at birth was 2500 g or less and non-LBW otherwise [26].

### 2.3. Assumed Causal Relationships

We used a casual mediation analysis to quantify BSE as a mediator in the relationship between IHFF and EBF outcomes. Figure 1 shows our model hypothesis of IHFF, EBF and BSE. IHFF (exposure) occurred earlier than BSE (mediator) and EBF (outcome), and BSE (mediator) did not occur later than EBF (outcome). The direct effect indicates the amount of influence of IHFF on EBF that does not pass through BSE (pathway b). The indirect effect represents a fraction of the total effect of IHFF on EBF that works through BSE (pathways a + c). (Figure 1)

### 2.4. Analysis

Our statistical analysis consisted of four parts. First, we examined the associations between IHFF, BSE, and EBF using logistic regression in both crude and adjusted models. Second, we used a causal mediation analysis to investigate whether BSE mediated the association between IHFF and EBF using the paramed command in Stata [27,28,29], a method used in previous studies [30]. In specific, we used a mediation analysis based on the counterfactual framework for causal inference [28,31]. Compared to the traditional approach, the counterfactual framework defines mediation effects in terms of counterfactuals in order to analyze variations in the occurrence of the study outcome alongside the exposure and potential mediator [31,32,33].

In our causal mediation analysis, we adopt the definitions and nomenclature of Pearl [34] for the risk difference scale and the extended concept of the odds ratio scale of Taylor [35]. Because we are examining how an exposure and a mediator interact, we decompose the total effect (TE) into natural direct (NDE) and natural indirect effects (NIE) using a logistic model [36]. The TE represents the odds ratio of EBF when comparing those exposed to IHFF with those who had not been exposed, while controlling for covariates. This allows the mediator (BSE) to vary naturally with exposure. The NDE captures the odds ratio for EBF when comparing those exposed to IHFF with those who had not been exposed, while keeping BSE at the level it would have been without IHFF exposure. The NIE calculates the odds ratio for EBF when comparing BSE under IHFF and not IHFF conditions, assuming the infant had received IHFF [35]. Finally, the odds ratio for the TE decomposes into a product of odds ratios for the NDE and the NIE [28,35]. Furthermore, we performed a subgroup analysis with parity and infant delivery. To carry this out, we divided the total sample into subgroups by parity and infant delivery and performed a causal mediation analysis for each group.

Fourth, we conducted sensitivity analyses to see if our results were replicated in subsamples of younger (0 to 2 months) and older (3 to 5 months) infants. Causal mediation is conducted under the assumption of no unmeasured confounding. The sensitivity analyses is to assess how robust the results are about direct and indirect effects to violations in the unmeasured confounder assumptions being made [37,38]. If the results were inconsistent, we repeated the causal mediation analyses of the final model, adjusting for the available covariates and infant age in months (younger vs. older) [30].

Statistical analyses were performed using Stata 16.0. Standard errors and corresponding 95% CIs (bias-corrected) were estimated using bootstrapping procedures with 1000 replications. The cutoff for 2-sided *p*-values was set at 0.05.

## 3. Results

### 3.1. Summary Statistics

Table 2 presents the summary statistics of the 778 participants. Among the infants in our sample, 34.1% were reported to have been exclusively breastfed. The majority of the sample (86.5%) had received IHFF. The infants were on average 2.8 months of age and nearly evenly distributed by sex. The majority of infants (over 80%) were delivered at full term, and 6.04% of them had a low birth weight. The sampled mothers were on average 28.6 years old; 39.2% of them had a high school degree or above; and 20.8% of them were employed. Additionally, 51.93% of the mothers had low breastfeeding self-efficacy and 72.1% of the mothers reported perceiving themselves to be in good health. More than half (57.2%) of the mothers had delivered their infant via caesarean section; 65.2% reported experiencing complications during pregnancy. Less than half (37.5%) of them were primiparous. Notably, 95.8% of the mothers reported experiencing breastfeeding difficulties.

We observed that IHFF was significantly more common after a mother delivered her infant via cesarean section (89.98%; 273/333) than vaginal delivery (81.98%; 400/445) (*p* < 0.01). Additionally, IHFF was more frequently observed when it was the mother’s first birth (90.75% (265/292) vs. 83.95% (408/486), *p* < 0.01).

### 3.2. Associations between IHFF, BSE, and EBF Outcomes

As shown by Table 3, we found that IHFF was significantly negatively associated with EBF (odds ratio, 0.41; 95% CI, 0.27 to 0.63). The association remained significant after covariate adjustment (odds ratio, 0.44; 95% CI, 0.23 to 0.83). Furthermore, IHFF was associated with lower BSE after a mother and infant had been discharged from the hospital (odds ratio, 0.50; 95% CI, 0.33 to 0.76). The significance and direction of the association also remained consistent in the adjusted model (odds ratio, 0.48; 95% CI, 0.31 to 0.74). Finally, post-hospital discharge, we found that higher BSE was associated with EBF (odds ratio, 3.67; 95% CI, 2.67 to 5.02). This association remained significant in the adjusted models (odds ratio, 3.90; 95% CI, 2.80 to 5.43).

### 3.3. Causal Mediation Analysis

The results of the causal mediation analysis are presented in Table 4. First, we found a significant TE (odds ratio, 0.47; 95% CI, 0.29 to 0.76) of IHFF on the likelihood of EBF after leaving the hospital. The NDE (odds ratio, 0.58; 95% CI, 0.36 to 0.94) and NIE (odds ratio, 0.81; 95% CI, 0.72 to 0.91) were also statistically significant, with 28% of the effect being mediated. This means that we found the association between IHFF and EBF. More specifically, infants who had received IHFF were only 47% as likely to be exclusively breastfed as infants who had not received IHFF. Notably, our results revealed that the decline in the EBF rate could potentially be mitigated by 28% if there was a change in BSE (i.e., shifting from a low level to a high level). In the subgroup analysis, there were no significant differences in the effects between parity subgroups, as well as between infant delivery subgroups (Table S1).

We then conducted sensitivity analyses across different infant age groups. From these analyses, we observed that the direction and significance of NIE was supported in two infant age groups: 0 to 2 months and 3 to 5 months (Table S2). However, the TE was significant only in infants aged 3 to 5 months and not for infants aged 0 to 2 months. To remove potential bias-associated age changes, we iterated the final analysis, adjusting for the available covariates and the age (0 = 0 to 2 months, 1 = 3 to 5 months), where the direction and significance of the associations remained the same as the results in Table 4.

## 4. Discussion

This study revealed that in-hospital formula feeding (IHFF) negatively impacted EBF outcomes, and that breastfeeding self-efficacy (BSE) mediated this association by 28%, as indicated by a significant NDE, NIE, and TE. We found that IHFF hindered later EBF and that BSE mediated this association.

First and foremost, we found that IHFF indeed increased the likelihood of an infant receiving non-EBF. This observation is supported by recent studies [11,39,40]. The direct link between IHFF and later EBF in these studies coincides with the results of our association analysis and causal mediation analysis, where both the TE and NDE were significant. This suggests that IHFF may interfere with the process of establishing EBF behavior. One potential explanation for IHFF’s negative impact on EBF is that IHFF affects a mother’s breast milk supply. Infants fed with formula rather than with maternal breastmilk post-birth tend to exhibit fewer instances of nipple suckling, resulting in a decrease in oxytocin production in mothers that subsequently leads to a reduction in their breast milk production [41,42,43]. Moreover, research has indicated that infants fed with formula tend to develop a preference for it, resulting in a resistance to consuming breast milk [12].

Second, we detected a negative association between IHFF and BSE. Previous research indicates that substituting breastfeeding with IHFF negatively affects a mother’s confidence in her ability to produce breastmilk; furthermore, the negative physiological effects of formula feeding on a mother’s breastmilk production may further exacerbate this loss of confidence [12,13]. Other research argues that certain mothers hold the belief that IHFF signifies a departure from pure breastfeeding, making EBF unachievable thereafter [44]. The odds ratio of associations of IHFF and BSE was less than 1, as was expected from the previous research [12], implying that IHFF was a risk factor for BSE.

Third, we found a positive association between BSE and EBF. Our results showed that mothers with high BSE were 3.9 times more likely to exclusively breastfeed their infant compared to mothers with low BSE. This is consistent with previous research findings that BSE predicts a mother’s choice to exclusively breastfeed and that interventions aimed at improving BSE can indirectly increase EBF rates [18,19].

The most important contribution of the current study is the finding that BSE is not only associated with both IHFF and EBF, but it also mediates the negative association between IHFF and EBF by 28%. Previous studies such as those by McCoy and Whipps have examined the relationship between IHFF and EBF, finding that IHFF reduces maternal confidence for breastfeeding and decreases the possibility of EBF [12,13]. However, none of these studies, to our knowledge, have examined the mediation role of “breastfeeding self-efficacy ” nor used mediation models to measure BSE’s impact. By employing a causal mediation analysis based on potential outcomes, we were able to demonstrate the likelihood that when an exposure is present, the outcome is mitigated through a mediator [35,45]. In our study, when IHFF occurs, the associated decline in EBF was reduced by 28% through the mediating role of BSE. Moreover, although BSE might not be the sole mediator in this relationship and there could be other pathways that this study did not measure or identify, this outcome still offers support for BSE as a potentially intervenable variable. Our study provides warnings and recommendations for hospitals to limit formula feeding during postpartum care. In cases when caregivers have already formula-fed in hospital, it may be a useful intervention to mitigate the negative impact of formula feeding in hospital on successful exclusive breastfeeding by increasing breastfeeding self-efficacy. Of course, further examination is needed to prove that this intervention is practicable.

The IHFF in our sample was high (86.5%). The inability of health workers to respond appropriately to questions about breastfeeding in the first few days of life, coupled with misunderstandings among caregivers, family members, or health workers about the multifactorial causes of infant behavior (e.g., crying), as well as the marketing of formula as solution to unsettled infants, may influence caregivers to choose formula [46]. Thus, in addition to improving the relevant knowledge and skills of health workers, it may be necessary to educate families about the right solution for dealing with unsettled infants and limit the marketing of infant formula.

This study has several strengths. First, a temporal sequence was established between exposure, mediator, and outcome variables. This aligns with the causal temporality criterion [24]. In our study, IHFF occurring earlier than EBF proved that IHFF could influence EBF, but not the other way around. Second, the causal mediation analyses allowed us to assess the mediating role of BSE between IHFF and EBF, which suggests potential intervention strategies.

However, we could not exclude the possibility of recall bias in using maternal recall to measure IHFF. Second, causal mediation analyses were based on the assumption that there were no unmeasured confounding variables [28]. Keeping this criterion in mind, we conducted sensitivity analyses and our results showed that the direction and significance of the causal mediation analyses results did not change even if unmeasured confounding variables were omitted.

## 5. Conclusions

In this study, IHFF was found to be negatively associated with EBF, with the association mediated by BSE. Findings of this study suggest that limiting IHFF and increasing mother’s BSE level are likely to improve EBF. Future research should explore the mechanisms underlying the association between IHFF, BSE, and EBF outcomes and clarify the nature of these associations. It is also important to develop interventions to reduce the potential risks inherent in IHFF and discover the protective effect of BSE on EBF. In addition, optimizing exclusive breastfeeding support and refusing of non-essential formula feeding in hospitals is important to hospital workers, researchers, parents, and infants themselves.

## Figures and Tables

**Table 1:** Breastfeeding Self-Efficacy Scale-Short Form (BSES-SF).

Items	Not Confident at All	Not Confident	Okay	Confident	Always Confident
Determine that my child is getting enough milk	1	2	3	4	5
Successfully cope with breastfeeding like I have with other challenging tasks	1	2	3	4	5
Breastfeed my child without using formula as a supplement	1	2	3	4	5
Ensure that my child is properly latched on for the whole feeding	1	2	3	4	5
Manage the breastfeeding situation to my satisfaction	1	2	3	4	5
Manage to breastfeed even if my child is crying	1	2	3	4	5
Keep wanting to breastfeed	1	2	3	4	5
Comfortably breastfeed with my family members present	1	2	3	4	5
Be satisfied with my breastfeeding experience	1	2	3	4	5
Deal with the fact that breastfeeding can be time-consuming	1	2	3	4	5
Finish feeding my child on one breast before switching to the other breast	1	2	3	4	5
Continue to breastfeed my child for every feeding	1	2	3	4	5
Manage to keep up with my child’s breastfeeding demands	1	2	3	4	5
Tell when my child is finished breastfeeding	1	2	3	4	5

**Table 2:** Summary statistics of study participants (N = 778).

	No. (%)	*p*-Value ^d^
IHFF		
no	105 (13.5)	-
yes	673 (86.5)	-
BSE **		0.001
low	404 (51.93)	
high	374 (48.07)	
EBF		<0.001
no	513 (65.94)	
yes	265 (34.06)	
Maternal age ^a^ (year)	28.6 (4.93)	0.375
Maternal education		0.267
junior high school and below	473 (60.8)	
high school and above	305 (39.2)	
Maternal perceived personal health		0.141
poor	217 (27.89)	
good	561 (72.11)	
Maternal occupation status		0.418
unemployed	616 (79.18)	
employed	162 (20.82)	
Family assets		0.98
poor	401 (51.54)	
good	377 (48.46)	
Infant delivery **		0.001
caesarean	445 (57.2)	
vaginal	333 (42.8)	
Infant age ^a^ (months)	2.77 (1.73)	0.4
Infant sex		0.397
male	393 (50.51)	
female	385 (49.49)	
Gestation		0.413
preterm	113 (14.52)	
full term	665 (85.48)	
Birth weight (g)		0.107
low birth weight	47 (6.04)	
non-low birth weight	731 (93.96)	
Breastfeeding difficulty ^b^		0.813
without difficulties	33 (4.24)	
with difficulties	745 (95.76)	
Pregnancy complications ^c^		0.571
no	271 (34.83)	
yes	507 (65.17)	
Parity **		0.007
multiparous	486 (62.47)	
primiparous	292 (37.53)	

IHFF, in-hospital formula feeding; BSE, breastfeeding self-efficacy; EBF, exclusive breastfeeding. ^a^ Mean(SD) ^b^ Breastfeeding difficulties include breast pains, back pains, baby having trouble sucking or latching on, sore or cracked or bleeding nipples, not enough breastmilk, doctors advising against breastfeeding, breast infection, clogged milk duct, breast engorgement, breasts leaked too much, taking too long for breast milk to come in, baby chocking when breastfeeding, baby cannot wake up on time to drink, baby is not interested in feeding or distracted, baby nursing too often, baby is not growing fast enough or is too underweight, and not enough time to feed child. ^c^ Pregnancy complications include gestational high blood pressure, gestational diabetes, gestational anemia, and any other pregnancy complications. ^d^ *p* value for difference between IHFF vs. non-IHFF. ** *p* < 0.01. Comparisons by chi-squared or *t*-test.

**Table 3:** Associations of in-hospital formula feeding and breastfeeding self-efficacy on exclusive breastfeeding outcomes (N = 778).

	EBF		BSE	
	OR	95% CI	OR	95% CI
**IHFF**				
Crude	0.41	0.27 to 0.63	0.50	0.33 to 0.76
Adjusted ^a^	0.44	0.29 to 0.68	0.48	0.31 to 0.74
**BSE ^b^**				
Crude	3.67	2.67 to 5.02		
Adjusted ^a^	3.90	2.80 to 5.43		

IHFF, in-hospital formula feeding; BSE, breastfeeding self-efficacy; EBF, exclusive breastfeeding; OR, odds ratio. ^a^ Maternal age, maternal education, maternal perceived personal health, maternal occupation status, family assets, infant delivery, infant sex, gestation, birth weight, breastfeeding difficulty, pregnancy complications, and parity were included as covariates in the adjusted model. ^b^ Breastfeeding self-efficacy was grouped into low and high breastfeeding self-efficacy according to p50.

**Table 4:** Causal mediation analyses showing the decomposition of associations of in-hospital formula feeding with exclusive breastfeeding outcomes. ^a^ (N = 778).

	OR (95% CI)
Total effect (TE ^b^)	0.47 (0.29 to 0.76)
Natural direct effect (NDE ^c^)	0.58 (0.36 to 0.94)
Natural indirect effect (NIE ^c^)	0.81 (0.72 to 0.91)
Percentage of indirect effect ^d^	28%

EBF, exclusive breastfeeding; OR, odds ratio; NDE, natural direct effect; NIE, natural indirect effect. ^a^ Breastfeeding self-efficacy was the mediating variable and grouped into low and high breastfeeding self-efficacy according to P50. ^b^ Maternal age, maternal education, maternal perceived personal health, maternal occupation status, assets, infant delivery, infant sex, gestation, birth weight, breastfeeding difficulty, pregnancy complications, and parity were included as covariates in the adjusted model. ^c^ NDE (bypassing breastfeeding self-efficacy after leaving the hospital) vs. NIE (via breastfeeding self-efficacy after leaving the hospital). The coefficients for NDE reflect the estimated difference between in-hospital formula feeding and those without in-hospital formula feeding given that breastfeeding self-efficacy was fixed at the natural level. The coefficients for NIE reflect the estimated difference between low breastfeeding self-efficacy and high breastfeeding self-efficacy that the exposure variable was fixed at in-hospital formula feeding. ^d^ Percentage of indirect effects = ln(ORNIE)/ln(ORTE).

## Data Availability

The data presented in this study are available upon request from the corresponding author. The data are not publicly available.

## Supplementary Materials

The following supporting information can be downloaded at: https://www.mdpi.com/article/10.3390/nu15245074/s1. Table S1: Causal mediation analyses stratified by parity and infant delivery; Table S2: Causal mediation sensitivity analyses across different infant age groups.
