# Supplementary material for: In-Hospital Formula Feeding Hindered Exclusive Breastfeeding: Breastfeeding Self-Efficacy as a Mediating Factor
Source: Nutrients. 2023 Dec 12;15(24):5074. doi: 10.3390/nu15245074 (PMC10746093; doi:10.3390/nu15245074)
Supplement: Supplementary file 1 [file nutrients-15-05074-s001.zip › nutrients-2733364-supplementary.pdf]

Table S1. Causal mediation analyses stratified by parity and infant delivery. [b]

	Subgroup analysis by parity, OR (95%CI)	
	primiparous (n = 292)	multiparous (n = 486)
Total effect (TE)	0.55 (0.22 to 1.41)	0.43 (0.24 to 0.77)
Natural direct effect (NDE [a])	0.61 (0.24 to 1.53)	0.55 (0.31 to 0.98)
Natural indirect effect (NIE [a])	0.91 (0.70 to 1.20)	0.79 (0.69 to 0.90)
P value for interaction [c]	0.990	
	**Subgroup analysis by infant delivery, OR (95%CI)**	
	caesarean (n = 445)	vaginal (n = 333)
Total effect (TE)	0.33 (0.15 to 0.70)	0.60 (0.31 to 1.17)
Natural direct effect (NDE)	0.37 (0.18 to 0.79)	0.79 (0.41 to 1.54)
Natural indirect effect (NIE)	0.88 (0.73 to 1.05)	0.76 (0.64 to 0.90)
P value for interaction	0.134	

OR, odds ratio [a] NDE (bypassing breastfeeding self-efficacy after leaving the hospital) vs NIE (via breastfeeding self-efficacy after leaving the hospital). The coefficients for NDE reflect the estimated difference between in-hospital formula feeding and those without in-hospital formula feeding given that breastfeeding self-efficacy was fixed at the natural level. The coefficients for NIE reflect the estimated difference between low breastfeeding self-efficacy and high breastfeeding self-efficacy that the exposure variable was fixed at in-hospital formula feeding. [b] Maternal age, maternal education, maternal perceived personal health, maternal occupation status, assets, infant delivery, infant sex, gestation, birth weight, breastfeeding difficulty, pregnancy complications and parity were included as covariates in the adjusted model. [c] Differences effect between subgroups were tested using the chow test.

Table S2. Causal mediation sensitivity analyses across different infant age groups

	**EBF, OR(95%CI)**		
	**0 to 2 months (n = 336)**	**3 to 5months (n = 442)**	**All participants (n = 778)**
Total effect	0.49(0.23 to 1.05)	**0.44(0.23 to 0.85)**	**0.46(0.28 to 0.75)**
Natural direct effect (NDE [a])	0.64(0.29 to 1.39)	0.53(0.28 to 1.01)	**0.57(0.35 to 0.93)**
Natural indirect effect (NIE [a])	**0.77(0.65 to 0.91)**	**0.84(0.71 to 0.98)**	**0.81(0.72 to 0.91)**

EBF, Exclusive breastfeeding; OR, odds ratio; NDE, natural direct effect; NIE, natural indirect effect. (1) Maternal age, maternal education, maternal perceived personal health, maternal occupation status, assets, infant delivery, infant sex, gestation, birth weight, breastfeeding difficulty, pregnancy complications and parity were included as covariates in the adjusted model. In all participants, months age was added. [a] NDE (bypassing breastfeeding self-efficacy

after leaving the hospital) vs NIE (via breastfeeding self-efficacy after leaving the hospital). The coefficients for NDE reflect the estimated difference between in-hospital formula feeding and those without in-hospital formula feeding given that breastfeeding self-efficacy was fixed at the natural level. The coefficients for NIE reflect the estimated difference between low breastfeeding self-efficacy and high breastfeeding self-efficacy that the exposure variable was fixed at in-hospital formula feeding.
